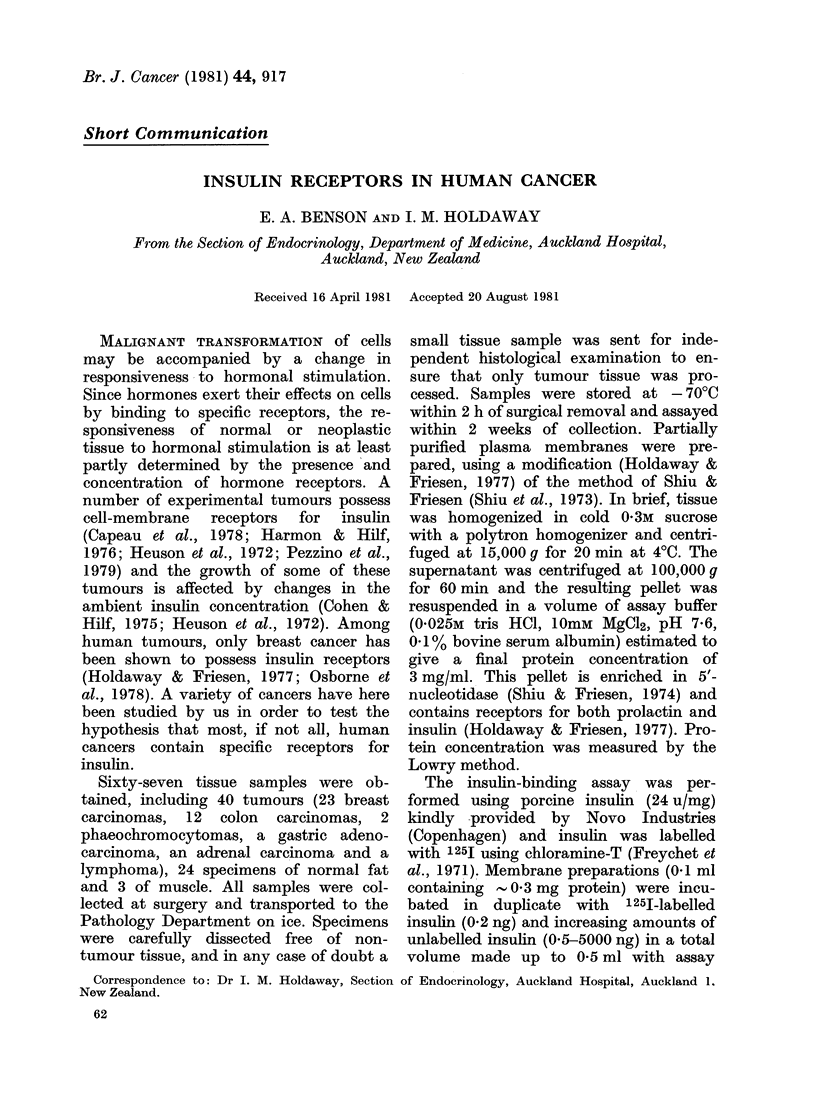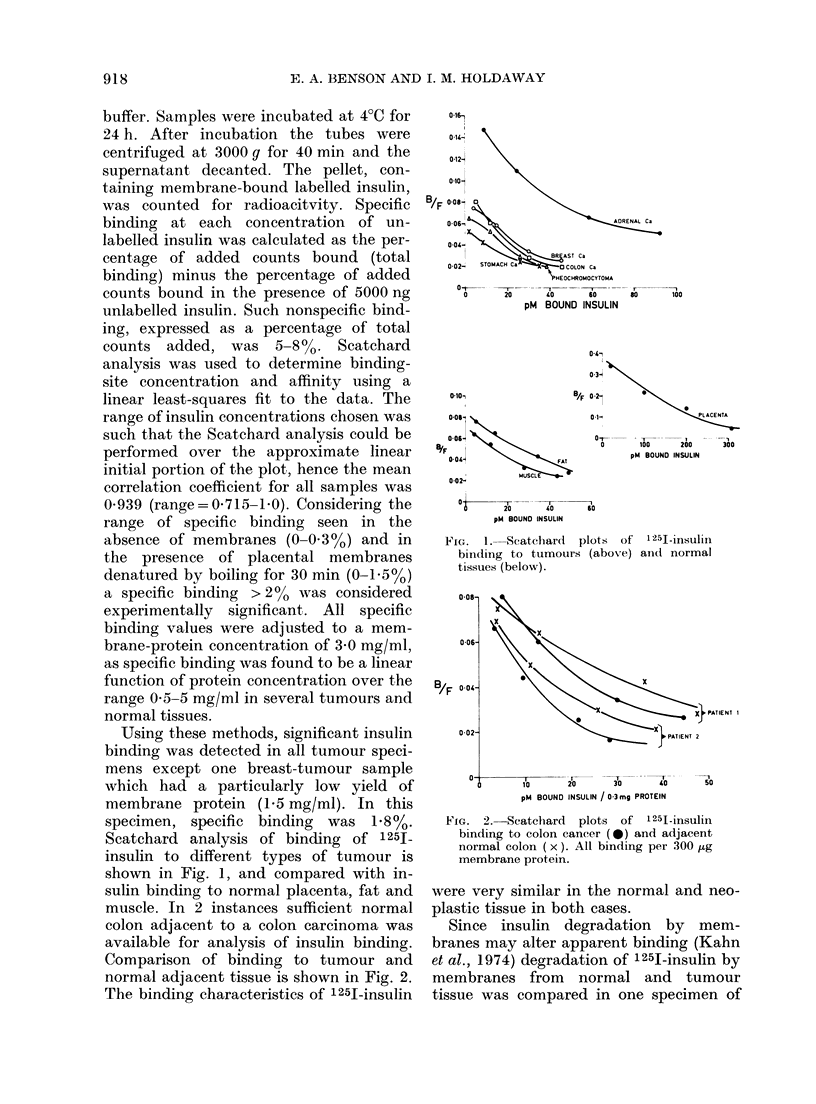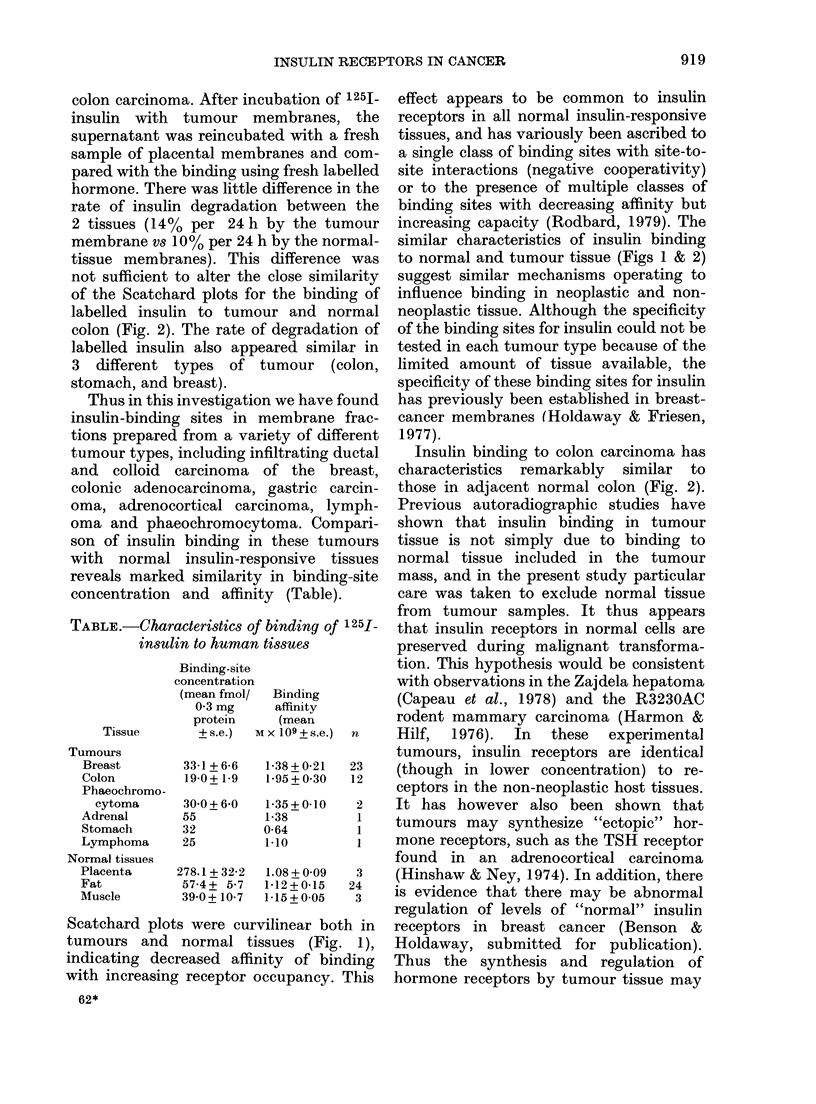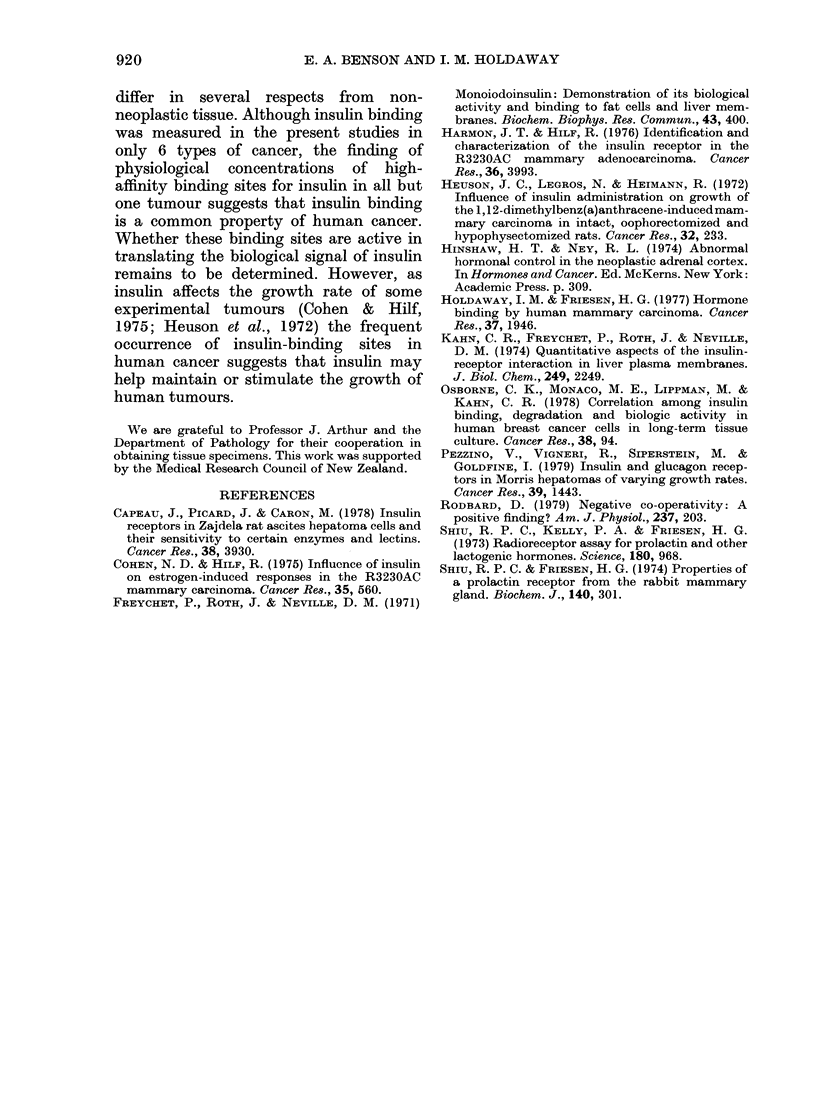# Insulin receptors in human cancer.

**DOI:** 10.1038/bjc.1981.292

**Published:** 1981-12

**Authors:** E. A. Benson, I. M. Holdaway


*Br. J. Cancer* (1981) **44**, 917

***Short Communication***

### INSULIN RECEPTORS IN HUMAN CANCER

E. A. BENSON AND I. M. HOLDAWAY

*From the Section of Endocrinology, Department of Medicine, Auckland Hospital, Auckland, New Zealand*

Received 16 April 1981 Accepted 20 August 1981

MALIGNANT TRANSFORMATION of cells may be accompanied by a change in responsiveness to hormonal stimulation. Since hormones exert their effects on cells by binding to specific receptors, the responsiveness of normal or neoplastic tissue to hormonal stimulation is at least partly determined by the presence and concentration of hormone receptors. A number of experimental tumours possess cell-membrane receptors for insulin (Capeau *et al.*, 1978; Harmon & Hilf, 1976; Heuson *et al.*, 1972; Pezzino *et al.*, 1979) and the growth of some of these tumours is affected by changes in the ambient insulin concentration (Cohen & Hilf, 1975; Heuson *et al.*, 1972). Among human tumours, only breast cancer has been shown to possess insulin receptors (Holdaway & Friesen, 1977; Osborne *et al.*, 1978). A variety of cancers have here been studied by us in order to test the hypothesis that most, if not all, human cancers contain specific receptors for insulin.

Sixty-seven tissue samples were obtained, including 40 tumours (23 breast carcinomas, 12 colon carcinomas, 2 phaeochromocytomas, a gastric adenocarcinoma, an adrenal carcinoma and a lymphoma), 24 specimens of normal fat and 3 of muscle. All samples were collected at surgery and transported to the Pathology Department on ice. Specimens were carefully dissected free of non-tumour tissue, and in any case of doubt a small tissue sample was sent for independent histological examination to ensure that only tumour tissue was processed. Samples were stored at −70°C within 2 h of surgical removal and assayed within 2 weeks of collection. Partially purified plasma membranes were prepared, using a modification (Holdaway & Friesen, 1977) of the method of Shiu & Friesen (Shiu *et al.*, 1973). In brief, tissue was homogenized in cold 0·3M sucrose with a polytron homogenizer and centrifuged at 15,000 *g* for 20 min at 4°C. The supernatant was centrifuged at 100,000 *g* for 60 min and the resulting pellet was resuspended in a volume of assay buffer (0·025M tris HCl, 10mM $MgCl_2$, pH 7·6, 0·1% bovine serum albumin) estimated to give a final protein concentration of 3 mg/ml. This pellet is enriched in 5′-nucleotidase (Shiu & Friesen, 1974) and contains receptors for both prolactin and insulin (Holdaway & Friesen, 1977). Protein concentration was measured by the Lowry method.

The insulin-binding assay was performed using porcine insulin (24 u/mg) kindly provided by Novo Industries (Copenhagen) and insulin was labelled with $^{125}I$ using chloramine-T (Freychet *et al.*, 1971). Membrane preparations (0·1 ml containing ~0·3 mg protein) were incubated in duplicate with $^{125}I$-labelled insulin (0·2 ng) and increasing amounts of unlabelled insulin (0·5–5000 ng) in a total volume made up to 0·5 ml with assay

Correspondence to: Dr I. M. Holdaway, Section of Endocrinology, Auckland Hospital, Auckland 1, New Zealand.

buffer. Samples were incubated at 4°C for 24 h. After incubation the tubes were centrifuged at 3000 *g* for 40 min and the supernatant decanted. The pellet, containing membrane-bound labelled insulin, was counted for radioacitvity. Specific binding at each concentration of unlabelled insulin was calculated as the percentage of added counts bound (total binding) minus the percentage of added counts bound in the presence of 5000 ng unlabelled insulin. Such nonspecific binding, expressed as a percentage of total counts added, was 5–8%. Scatchard analysis was used to determine binding-site concentration and affinity using a linear least-squares fit to the data. The range of insulin concentrations chosen was such that the Scatchard analysis could be performed over the approximate linear initial portion of the plot, hence the mean correlation coefficient for all samples was 0·939 (range = 0·715–1·0). Considering the range of specific binding seen in the absence of membranes (0–0·3%) and in the presence of placental membranes denatured by boiling for 30 min (0–1·5%) a specific binding > 2% was considered experimentally significant. All specific binding values were adjusted to a membrane-protein concentration of 3·0 mg/ml, as specific binding was found to be a linear function of protein concentration over the range 0·5–5 mg/ml in several tumours and normal tissues.

Using these methods, significant insulin binding was detected in all tumour specimens except one breast-tumour sample which had a particularly low yield of membrane protein (1·5 mg/ml). In this specimen, specific binding was 1·8%. Scatchard analysis of binding of $^{125}$I-insulin to different types of tumour is shown in Fig. 1, and compared with insulin binding to normal placenta, fat and muscle. In 2 instances sufficient normal colon adjacent to a colon carcinoma was available for analysis of insulin binding. Comparison of binding to tumour and normal adjacent tissue is shown in Fig. 2. The binding characteristics of $^{125}$I-insulin

FIG. 1.—Scatchard plots of $^{125}$I-insulin binding to tumours (above) and normal tissues (below).

FIG. 2.—Scatchard plots of $^{125}$I-insulin binding to colon cancer (●) and adjacent normal colon (×). All binding per 300 μg membrane protein.

were very similar in the normal and neoplastic tissue in both cases.

Since insulin degradation by membranes may alter apparent binding (Kahn *et al.*, 1974) degradation of $^{125}$I-insulin by membranes from normal and tumour tissue was compared in one specimen of colon carcinoma. After incubation of $^{125}$I-insulin with tumour membranes, the supernatant was reincubated with a fresh sample of placental membranes and compared with the binding using fresh labelled hormone. There was little difference in the rate of insulin degradation between the 2 tissues (14% per 24 h by the tumour membrane *vs* 10% per 24 h by the normal-tissue membranes). This difference was not sufficient to alter the close similarity of the Scatchard plots for the binding of labelled insulin to tumour and normal colon (Fig. 2). The rate of degradation of labelled insulin also appeared similar in 3 different types of tumour (colon, stomach, and breast).

Thus in this investigation we have found insulin-binding sites in membrane fractions prepared from a variety of different tumour types, including infiltrating ductal and colloid carcinoma of the breast, colonic adenocarcinoma, gastric carcinoma, adrenocortical carcinoma, lymphoma and phaeochromocytoma. Comparison of insulin binding in these tumours with normal insulin-responsive tissues reveals marked similarity in binding-site concentration and affinity (Table).

TABLE.—*Characteristics of binding of $^{125}$I-insulin to human tissues*

| Tissue | Binding-site concentration (mean fmol/ 0·3 mg protein ± s.e.) | Binding affinity (mean M × 10$^9$ ± s.e.) | *n* |
|---|---|---|---|
| Tumours | | | |
| Breast | 33·1 ± 6·6 | 1·38 ± 0·21 | 23 |
| Colon | 19·0 ± 1·9 | 1·95 ± 0·30 | 12 |
| Phaeochromocytoma | 30·0 ± 6·0 | 1·35 ± 0·10 | 2 |
| Adrenal | 55 | 1·38 | 1 |
| Stomach | 32 | 0·64 | 1 |
| Lymphoma | 25 | 1·10 | 1 |
| Normal tissues | | | |
| Placenta | 278.1 ± 32·2 | 1.08 ± 0·09 | 3 |
| Fat | 57·4 ± 5·7 | 1·12 ± 0·15 | 24 |
| Muscle | 39·0 ± 10·7 | 1·15 ± 0·05 | 3 |

Scatchard plots were curvilinear both in tumours and normal tissues (Fig. 1), indicating decreased affinity of binding with increasing receptor occupancy. This effect appears to be common to insulin receptors in all normal insulin-responsive tissues, and has variously been ascribed to a single class of binding sites with site-to-site interactions (negative cooperativity) or to the presence of multiple classes of binding sites with decreasing affinity but increasing capacity (Rodbard, 1979). The similar characteristics of insulin binding to normal and tumour tissue (Figs 1 & 2) suggest similar mechanisms operating to influence binding in neoplastic and non-neoplastic tissue. Although the specificity of the binding sites for insulin could not be tested in each tumour type because of the limited amount of tissue available, the specificity of these binding sites for insulin has previously been established in breast-cancer membranes (Holdaway & Friesen, 1977).

Insulin binding to colon carcinoma has characteristics remarkably similar to those in adjacent normal colon (Fig. 2). Previous autoradiographic studies have shown that insulin binding in tumour tissue is not simply due to binding to normal tissue included in the tumour mass, and in the present study particular care was taken to exclude normal tissue from tumour samples. It thus appears that insulin receptors in normal cells are preserved during malignant transformation. This hypothesis would be consistent with observations in the Zajdela hepatoma (Capeau *et al.*, 1978) and the R3230AC rodent mammary carcinoma (Harmon & Hilf, 1976). In these experimental tumours, insulin receptors are identical (though in lower concentration) to receptors in the non-neoplastic host tissues. It has however also been shown that tumours may synthesize "ectopic" hormone receptors, such as the TSH receptor found in an adrenocortical carcinoma (Hinshaw & Ney, 1974). In addition, there is evidence that there may be abnormal regulation of levels of "normal" insulin receptors in breast cancer (Benson & Holdaway, submitted for publication). Thus the synthesis and regulation of hormone receptors by tumour tissue may differ in several respects from non-neoplastic tissue. Although insulin binding was measured in the present studies in only 6 types of cancer, the finding of physiological concentrations of high-affinity binding sites for insulin in all but one tumour suggests that insulin binding is a common property of human cancer. Whether these binding sites are active in translating the biological signal of insulin remains to be determined. However, as insulin affects the growth rate of some experimental tumours (Cohen & Hilf, 1975; Heuson *et al.*, 1972) the frequent occurrence of insulin-binding sites in human cancer suggests that insulin may help maintain or stimulate the growth of human tumours.

We are grateful to Professor J. Arthur and the Department of Pathology for their cooperation in obtaining tissue specimens. This work was supported by the Medical Research Council of New Zealand.

### REFERENCES

CAPEAU, J., PICARD, J. & CARON, M. (1978) Insulin receptors in Zajdela rat ascites hepatoma cells and their sensitivity to certain enzymes and lectins. *Cancer Res.*, **38**, 3930.

COHEN, N. D. & HILF, R. (1975) Influence of insulin on estrogen-induced responses in the R3230AC mammary carcinoma. *Cancer Res.*, **35**, 560.

FREYCHET, P., ROTH, J. & NEVILLE, D. M. (1971) Monoiodoinsulin: Demonstration of its biological activity and binding to fat cells and liver membranes. *Biochem. Biophys. Res. Commun.*, **43**, 400.

HARMON, J. T. & HILF, R. (1976) Identification and characterization of the insulin receptor in the R3230AC mammary adenocarcinoma. *Cancer Res.*, **36**, 3993.

HEUSON, J. C., LEGROS, N. & HEIMANN, R. (1972) Influence of insulin administration on growth of the 1,12-dimethylbenz(a)anthracene-induced mammary carcinoma in intact, oophorectomized and hypophysectomized rats. *Cancer Res.*, **32**, 233.

HINSHAW, H. T. & NEY, R. L. (1974) Abnormal hormonal control in the neoplastic adrenal cortex. In *Hormones and Cancer*. Ed. McKerns. New York: Academic Press. p. 309.

HOLDAWAY, I. M. & FRIESEN, H. G. (1977) Hormone binding by human mammary carcinoma. *Cancer Res.*, **37**, 1946.

KAHN, C. R., FREYCHET, P., ROTH, J. & NEVILLE, D. M. (1974) Quantitative aspects of the insulin-receptor interaction in liver plasma membranes. *J. Biol. Chem.*, **249**, 2249.

OSBORNE, C. K., MONACO, M. E., LIPPMAN, M. & KAHN, C. R. (1978) Correlation among insulin binding, degradation and biologic activity in human breast cancer cells in long-term tissue culture. *Cancer Res.*, **38**, 94.

PEZZINO, V., VIGNERI, R., SIPERSTEIN, M. & GOLDFINE, I. (1979) Insulin and glucagon receptors in Morris hepatomas of varying growth rates. *Cancer Res.*, **39**, 1443.

RODBARD, D. (1979) Negative co-operativity: A positive finding? *Am. J. Physiol.*, **237**, 203.

SHIU, R. P. C., KELLY, P. A. & FRIESEN, H. G. (1973) Radioreceptor assay for prolactin and other lactogenic hormones. *Science*, **180**, 968.

SHIU, R. P. C. & FRIESEN, H. G. (1974) Properties of a prolactin receptor from the rabbit mammary gland. *Biochem. J.*, **140**, 301.